# Lipophilic Allergens, Different Modes of Allergen-Lipid Interaction and Their Impact on Asthma and Allergy

**DOI:** 10.3389/fimmu.2019.00122

**Published:** 2019-02-14

**Authors:** Uta Jappe, Christian Schwager, Andra B. Schromm, Nestor González Roldán, Karina Stein, Holger Heine, Katarzyna A. Duda

**Affiliations:** ^1^Division of Clinical and Molecular Allergology, Research Center Borstel, Leibniz Lung Center, Airway Research Center North, German Center for Lung Research, Borstel, Germany; ^2^Interdisciplinary Allergy Outpatient Clinic, Department of Pneumology, University of Luebeck, Borstel, Germany; ^3^Division of Immunobiophysics, Research Center Borstel, Leibniz Lung Center, Borstel, Germany; ^4^Junior Research Group of Allergobiochemistry, Research Center Borstel, Leibniz Lung Center, Airway Research Center North, German Center for Lung Research, Borstel, Germany; ^5^Division of Innate Immunity, Research Center Borstel, Leibniz Lung Center, Airway Research Center North, German Center for Lung Research, Borstel, Germany

**Keywords:** asthma, food allergy, house dust mite, innate immunity, lipophilic allergens, lipids, peanut, pulmonary surfactants

## Abstract

Molecular allergology research has provided valuable information on the structure and function of single allergenic molecules. There are several allergens in food and inhalant allergen sources that are able to interact with lipid ligands *via* different structural features: hydrophobic pockets, hydrophobic cavities, or specialized domains. For only a few of these allergens information on their associated ligands is already available. Several of the allergens are clinically relevant, so that it is highly probable that the individual structural features with which they interact with lipids have a direct effect on their allergenic potential, and thus on allergy development. There is some evidence for a protective effect of lipids delaying the enzymatic digestion of the peanut (*Arachis hypogaea*) allergen Ara h 8 (hydrophobic pocket), probably allowing this molecule to get to the intestinal immune system intact (sensitization). Oleosins from different food allergen sources are part of lipid storage organelles and potential marker allergens for the severity of the allergic reaction. House dust mite (HDM), is more often associated with allergic asthma than other sources of inhalant allergens. In particular, lipid-associated allergens from *Dermatophagoides pteronyssinus* which are Der p 2, Der p 5, Der p 7, Der p 13, Der p 14, and Der p 21 have been reported to be associated with severe allergic reactions and respiratory symptoms such as asthma. The exact mechanism of interaction of these allergens with lipids still has to be elucidated. Apart from single allergens glycolipids have been shown to directly induce allergic inflammation. Several—in parts conflicting—data exist on the lipid (and allergen) and toll-like receptor interactions. For only few single allergens mechanistic studies were performed on their interaction with the air-liquid interface of the lungs, in particular with the surfactant components SP-A and SP-D. The increasing knowledge on protein-lipid-interaction for lipophilic and hydrophobic food and inhalant allergens on the basis of their particular structure, of their capacity to be integral part of membranes (like the oleosins), and their ability to interact with membranes, surfactant components, and transport lipids (like the lipid transfer proteins) are essential to eventually clarify allergy and asthma development.

## Introduction

In recent decades, allergies have become the number one chronic disease in many parts of the world affecting up to 30% of the population in each country (1). Therefore, much effort has been devoted to identifying and characterizing single allergens in order to improve routine diagnostic tests and offer therapeutic interventions. Until lately, however, this work was dedicated to water-soluble allergens because due to their hydrophobic/lipophilic properties lipophilic allergens were not isolated by conventional extraction procedures and were not encountered in IgE screening experiments. The recent discovery of a group of unique lipophilic allergens, termed oleosins, in food allergen sources and their association with severe allergic reactions demonstrated the importance of non-water-soluble allergens and gave impetus to intensify studies on allergen-lipid-interaction and how it impacts the allergic sensitization process (2–4). This review will give an overview of different groups of lipophilic/lipid-associated allergens, the mechanisms of allergen-lipid-interaction known so far and discuss its immunological impact on disease development (Table 1). The focus will be on the two most common clinically relevant sources of lipophilic allergens, house dust mite, and peanut.

**Table 1:** Allergens and their interaction with lipids.

**Protein family**	**Source**	**Allergen**	**Allergological relevance**	**Mode of lipid/ligand interaction**	**Resulting effects**
Bet v 1 like	Birch *(Betula verrucosa)*	Bet v 1	Major allergen, associated with mild allergic reactions	Binds ligands *via* hydrophobic pocket	Binds and permeabilizes membranes
	Peanut *(Arachis hypogaea)*	Ara h 8	Minor allergen, associated with mild allergic reactions, marker for pollen-associated food allergy	Binds lipids *via* hydrophobic pocket	Delayed enzymatic digestion (see Figure 1B), increased thermal stability, enhanced uptake in intestinal mucosa
Non-specific lipid transfer protein	Peach *(Prunus persica)*	Pru p 3	Pan-allergen, associated with severe allergic reactions (Mediterranean area)	Binds fatty acids in inner hydrophobic cavity	Induction of conformational changes that lead to increased IgE-binding (see Figure 1C)
	Peanut *(Arachis hypogaea)*	Ara h 9	Pan-allergen, associated with severe allergic reactions (Mediterranean area)	Potentially binds lipids, phospholipids in inner hydrophobic cavity	Unknown
	Grape *(Vitas vinifera)*	Vit v1	Pan-allergen, associated with severe allergic reactions (Mediterranean area)	binds phosphatidylcholine	Delayed enzymatic digestion
Globulin	Peanut *(Arachis hypogaea)*	Ara h 1	Major allergen, associated with severe allergic reactions	Interaction with phosphatidylglycerol vesicles	Delayed enzymatic digestion
	Mustard *(Sinapis alba)*	Sin a 2	Major allergen, associated with severe allergic reactions	Interaction with phosphatidylglycerol vesicles and mustard lipids	Protection against enzymatic digestion & microsomal degradation, activation of human DCs
2S Albumin	Brazil nut *(Bertholletia excelsa)*	Ber e 1	Major allergen, potentially associated with severe allergic reactions	Lipid-binding hydrophobic cavity is assumed	Co-administration with brazil nut lipids induced IgE and IgG1-response in mice and IL-4 in murine and human CD1d-restricted iNKT cells
	Peanut *(Arachis hypogaea)*	Ara h 2	Major allergen, associated with severe allergic reactions (marker allergen)	None	Might inhibit tryptic degradation of co-administered peanut allergens
Oleosins	Peanut *(Arachis hypogaea)*	Ara h 10	Potential major allergens, associated with severe allergic reactions (potential marker allergens)	Bind phospholipids and lipids *via* hydrophobic domain creating an oil body	Potentially enhanced uptake of oil bodies *via* lipid-carrier-mediated transport mechanism (see Figure 1E)
		Ara h 11			
		Ara h 14			
		Ara h 15			
	Sesame *(Sesamum indicum)*	Ses i 4 Ses i 5	Potential major allergens, associated with severe allergic reactions (potential marker allergens)	Bind phospholipids and lipids *via* hydrophobic domain creating an oil body	
	Hazelnut *(Corylus avellana)*	Cor a 12			
		Cor a 13			
Lipocalin	Cow's milk *(Bos domesticus)*	Bos d 5	Major allergen	Carries hydrophobic molecules, phosphatidylcholine	Insertion into bilayers, protection against enzymatic digestion
	Dog *(Canis familiaris)*	Can f 6	Unknown	Binds LPS	Enhancement of LPS/TLR4-signaling (see Figure 1D)
Secreto-globulin	Cat *(Felis domesticus)*	Fel d 1	Major allergen	Potentially binds TLR-ligands	Enhancement of TLR2 and TLR4 signaling
Group 2 mite allergen	House dust mite *(Dermatophagoides pteronyssinus)*	Der p 2	Major allergen, more often recognized by asthmatics	Binds LPS due to structural similarity with MD-2	Enhancement of LPS/TLR4-signaling (see Figure 1D) resulting in airway Th2 inflammation
	House dust mite *(Dermatophagoides farinae)*	Der f 2	Major allergen, more often recognized by asthmatics	Binds LPS due to structural similarity with MD-2	
Group 5/7 mite allergen	House dust mite *(Dermatophagoides pteronyssinus)*	Der p5	Minor allergen, more often recognized by asthmatics	Hydrophobic cavities that might bind apolar ligands	Potential stimulation of TLR2 (see Figure 1D)
		Der p 7	Minor allergen, more often recognized by asthmatics	Hydrophobic cavities that might bind apolar ligands	
Group 13 mite allergen	House dust mite *(Dermatophagoides pteronyssinus)*	Der p 13	Minor allergen	Selective binding of fatty acids in inner cavity	Induction of airway epithelial cell activation through TLR2-MyD88-NF-κB and MAPK-dependent mechanisms (see Figure 1D)
Group 14 mite allergen	House dust mite *(Dermatophagoides pteronyssinus)*	Der p 14	Minor allergen	Potential transporter of lipids	Unknown
Group 21 mite allergen	House dust mite *(Dermatophagoides pteronyssinus)*	Der p 21	Minor allergen	Potentially binds lipids from house dust mite	Activation of airway epithelial cells through TLR2 signaling

*Major allergen: recognized by >50% of individuals allergic to the culprit allergen source. Minor allergen: recognized by <50% of individuals allergic to the culprit allergen source*.

## House Dust Mite and Peanut As Model for Allergen-Lipid-Interaction

House dust mites (HDM) are the major allergen source found in house dust and a common elicitor of severe respiratory symptoms such as asthma. Surprisingly, more than 80% of individuals suffering from asthma are allergic to HDM (5). This finding triggered an increased interest in the identification of allergens from the two most relevant mite species, the American house dust mite (*Dermatophagoides farinae*, Der f) and the European house dust mite (*Dermatophagoides pteronyssinus*, Der p). Until now, more than 20 allergens have been identified for each species (Der f 1–36; Der p 1–37) (www.allergen.org), some of which with lipophilic properties. Interestingly, the lipophilic allergens have been more often associated with asthmatic diseases (6, 7).

**Figure 1:**
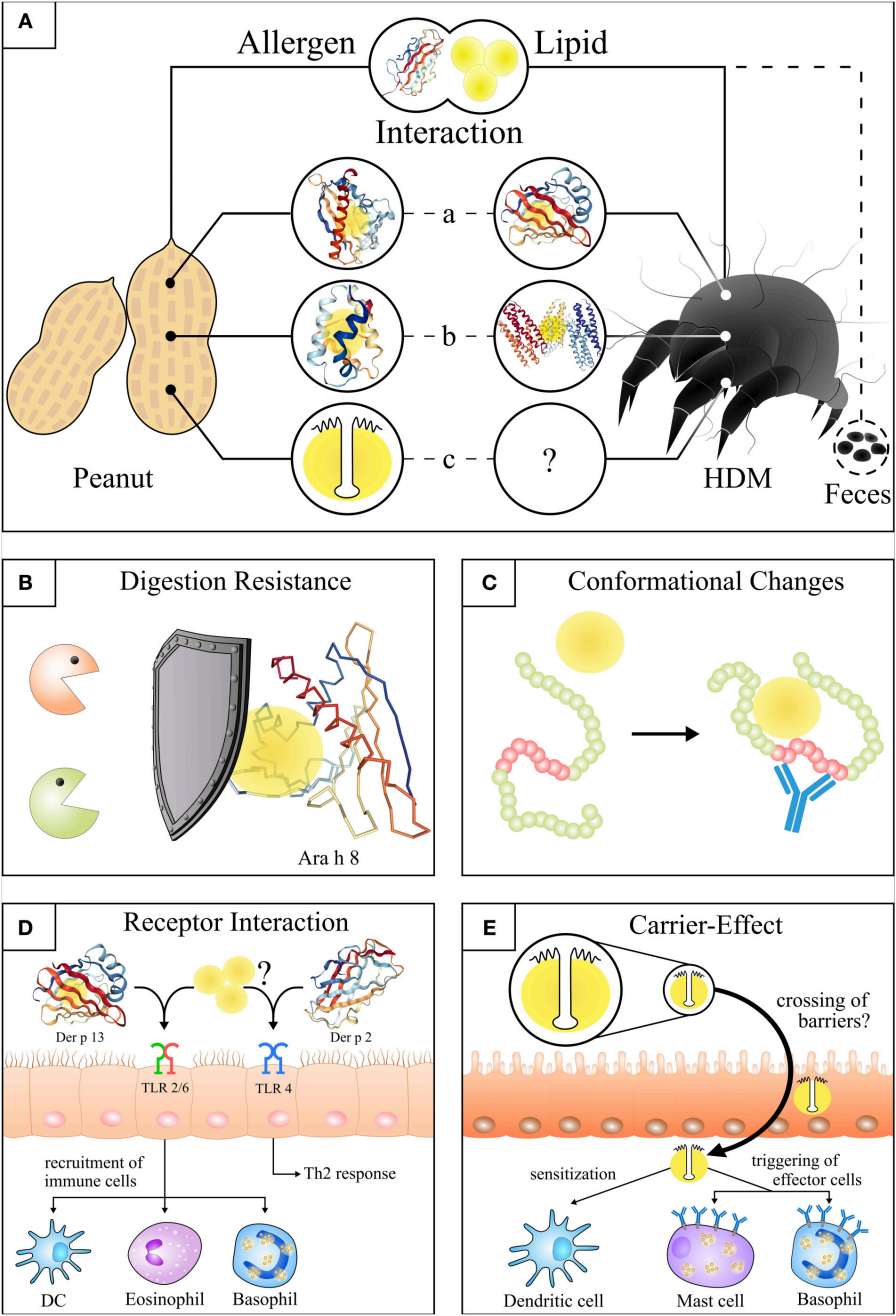
Structures and effects of the allergen-lipid-interaction in peanut and house dust mite. **(A)** Illustration of the molecular interaction of lipids and allergens from peanut and house dust mite. (a) Lipids bound to the hydrophobic pocket of Ara h 8^*^ (14) (PDB ID: 4M9B) and Der p 13^**^. (b) Lipids integrated into the hydrophobic cavity of Ara h 9^***^ and Der p 5 (15) (PDB ID: 4M9B). (c) Lipids attached to the hydrophobic domain of peanut oleosins^****^. **(B)** Attached lipids delay or prevent the digestion of lipophilic proteins (simplified cartoon: lipids bound to Ara h 8). **(C)** Lipid binding induces conformational changes of allergens that lead to the exposure of hidden epitopes (red part). **(D)** Potential initiation of HDM sensitization through activation of TLR2 by Der p 13^**^ and associated or free lipids (left side) or Der p 2 (PDB ID: 1KTJ) mediated TLR4 activation in bronchial epithelial cells (right side). **(E)** Uptake of oil bodies^****^ (and intrinsic proteins e.g. oleosins) by a lipid carrier-mediated transport in the gut as a potential route of sensitization. The individual structures of proteins were visualized by use of NGL viewer (16). ^*^Lipids were integrated according to the analogous protein Bet v 1 (17) (PDB ID: 4A83). ^**^Protein structure of the Der p 13 homologues Der f 13 (18) (PDB ID: 2a0a) is shown as there is no PDB structure of Der p 13 available. ^***^For visualization of the non-specific lipid transfer protein (nsLTP, Ara h 9) the structure of the similar nsLTP from maize (19) (PDB ID: 1MZM) was used. ^****^Simplification of a peanut oil body. A more realistic structure can be looked up at Jappe and Schwager (20).

Peanut is an important source of nutritionally valuable lipids (fat content ~50%). However, it is also one of the most potent allergen sources and a major cause of food-induced anaphylaxis in industrialized countries (8–10). Therefore, peanut allergens have been intensively studied to identify those important for diagnosis and therapy. Among the 16 officially registered peanut allergens (www.allergen.org) two have been identified by us to be associated with lipids, Ara h 8 and Ara h 9 (11, 12). Nevertheless, the accompanying lipids did neither effect their extractability nor their IgE reactivity in immunological test systems, thus their role in peanut allergy did not become apparent at that time. Over the last years the view on lipid-associated allergens has changed, in particular by the discovery of the oleosins, a unique group of water-insoluble membrane proteins. They are absent from diagnostic extracts but have been shown to be potential marker allergens for the severity of the allergic reaction to food (2, 3, 13). These findings have directed our research to the question of the effect of lipids and their association with lipophilic allergens in the context of allergic diseases (see Figure 1).

Lipids are small hydrophobic or amphipathic molecules which, according to the International Lipid Classification and Nomenclature Committee, are categorized into 8 groups based on their structural features. These groups are fatty acyls, glycerolipids, sterol lipids, glycerophospholipids, sphingolipids, prenol lipids, saccharolipids, and polyketides (21).

Apart from the lipids interacting directly with allergens, there are also various lipids co-delivered with allergens. They originate either from the allergen source *per se* or from microbes associated to pollen or house dust mites [reviewed by (22)]. Lipids, as part of the allergen source, occur in pollen coats [so called pollenkit, where they exhibit protective functions for the plants (23)], in matrices of plant and animal foods and in animal dander. These lipids can modulate the immune system by interacting with innate lymphocytes, such as NKT cells (24–26). Examples of such immunomodulatory lipids are lipopolysaccharide (LPS) and lipid mediators, i.e., Pollen Associated Lipid Mediators (PALMs).

There is more detailed knowledge on the protein than on the lipid molecules when considering structural interaction or the immunological effect on disease pathomechanisms.

There are several examples in food as well as inhalant allergen sources for structural allergen-lipid-associations, which have in parts already been elucidated, structurally, and/or immunologically. Since they have been already summarized (22, 27), in our review we focus on their potential clinical relevance.

## Allergens From Different Sources (Food, Mammals, Arthropods), and Their Association With Lipids

In order to understand the effect of structure on the allergenicity of certain allergen sources it is important to understand essential definitions of molecular allergology.

The official nomenclature of single allergens consists of the abbreviated Latin name of the allergen source [the first 3 or 4 letters of the genus, i.e., *Betula* (birch)], the first or the first 2 letters of the species (*verrucosa*) and, in general, the number following the chronological order in which they were identified, (i.e., Bet v 1) (28). Basically, single peanut allergens are named *Arachis hypogaea* (Ara h) 1–17; house dust mite allergens (*Dermatophagoides pteronyssinus* (Der p 1–37), *D. farinae* (Der f 1–36). A huge number of allergens are allocated to only few protein families. Exemplified for peanut, these are the following: the Bet v 1 (*Betula verrucosa*) superfamily, the prolamin superfamily (that includes the lipid-transfer proteins), the conglutin-like storage proteins; 2S-albumins, vicilin-like storage proteins (7S-globulin), the defensins (29), and the oleosins (4). The association with lipids has been described for the allergen families Bet v 1-superfamily, lipid transfer proteins, 2S albumins, 7 and 11S globulins, oleosins, lipocalins, apolipophorins, and the mite allergen groups 2, 5, and 7.

The respective proteins possess hydrophobic / lipophilic properties which provide the prerequisites for allergen-lipid-interactions either *via* binding through hydrophobic cavities (15, 30–32), ionic (33), or hydrophobic bonds (34).

These intrinsic properties of the allergens most probably have an impact on their allergenicity. Basically, allergens can carry lipids (“lipid cargo”) (27), and these lipids can alter the allergenicity of allergens by modifying their structure and biochemical properties. On the other hand, it is most plausible that lipids are carriers for allergens (see oil bodies-oleosins). The structural prerequisites are different and only partly elucidated. Some lipids change the tertiary structure of proteins so that allergenic epitopes are exposed to IgE antibodies (see paragraph heading Lipid Transfer Proteins) (see Figures 1A–D).

## Food Allergen Sources

### Bet v 1 and Its Homologs in Food Allergen Sources

It was shown that the Bet v 1-molecule has a hydrophobic pocket binding various physiologically important lipophilic ligands, including free fatty acids (30, 35, 36). We could show that the Bet v 1-homolog of peanut and relevant marker allergen for pollen-associated food (class II) allergy, Ara h 8, purified from roasted peanuts, possesses a hydrophobic pocket where lipids are attached to the protein (12). This is noteworthy as there is some evidence for a protective effect of lipids delaying the enzymatic digestion and supporting the uptake of allergens by intestinal mucosa cells (see Figure 1B) (12, 22).

### Lipid Transfer Proteins (LTP)

Nonspecific lipid transfer proteins (nsLTP) are a class of proteins with potent allergenic representatives in pollen and food allergen sources. Basically, characteristic features of LTPs are a low molecular weight of ~10 kDa, and a hydrophobic cavity which pervades the molecule and allows the hosting of ligands such as fatty acids. In the Mediterranean area LTP-sensitization is associated with severe allergic reactions (11). However, some single cases occur in Northern Europe as well (37), and their number is increasing. Peanut possesses three lipid transfer proteins. The first one is Ara h 9 (with two isoforms) (11), to which 38.5% of peanut-allergic individuals in the Mediterranean area were sensitized, whereas in the same population IgE-reactivity to the storage proteins and major allergens Ara 1, Ara h 2, and Ara h 3 was altogether 4.8%. Further, there are Ara h 16 and Ara h 17, for which the WHO/IUIS^1^ allergen nomenclature documentation exists but no publications (www.allergen.org). For Ara h 9, Ara h 16, and Ara h 17 the lipid-association has not yet been elucidated in contrast to other members of this protein family. However, Krause et al. had evidence that the peach allergen *Prunus persica* 3 (Pru p 3) or Ara h 9 may be the primary sensitizing allergens in those cases where peanut storage proteins or Ara h 8 were not responsible for primary sensitization to peanuts. That makes Ara h 9 an important addition to the component-resolved diagnosis. Furthermore, a deeper insight into its sensitization route could provide important information to develop methods for the prevention of food allergy (11). The 3-dimensional structure is very similar among non-specific (ns) LTPs due to characteristic disulfide bonds (11). Sequence identity among LTPs is generally low with the exception of Pru p 3 and *Malus domesticus* (Mal d) 3 (apple). However, nsLTPs from various allergen sources may differ considerably with regard to their potential allergen cross-reactivity.

A detailed review on LTPs was published in the special issue of Frontiers in Immunology, Role of Lipids in the Dynamics of Allergic Airway Inflammation by Scheurer and Schülke (38). We chose Pru p 3 to include an exemplified report on LTPs in the context of our own review. Pru p 3 has been investigated in detail for its lipid-association, is considered the clinically most important and best characterized food LTP and a marker allergen for LTP-sensitization (39). For peach and hazelnut LTPs investigations with and without lipids have been performed, suggesting that the binding of lipophilic ligands altered the cavity (“structural plasticity”) (40). Dubiela et al. have reported the binding of the following substances to Pru p 3: lauric acid, cis-parinaric acid, palmitic acid, and linoleic acid. The authors investigated whether peach-LTP-ligand (lipid) interaction affected IgE-binding in sera from peach allergic patients. It was shown that the region most probably affected by the structural plasticity and the conformational changes of the hydrophobic cavity induced by oleic acid binding contained the major IgE epitope responsible for severe reactions. Interestingly, this fatty acid also bound recombinant Pru p 3 which is in contrast to our own observations for the Bet v 1-homolog in peanut, the Ara h 8 (12). However, it was shown that the preincubation of recombinant (r) Pru p 3 with oleic acid increased the IgE-reactivity of the sera of 10 peach allergic patients in an ELISA when compared with rPru p 3 alone. The next step was a cellular diagnostic test, the basophil activation test, which confirmed the ELISA results: rPru p 3 plus oleic acid increased the number of activated basophils when compared with rPru p 3 alone (40).

Similar results were obtained with a grape *Vitas vinifera* nsLTP (Vit v 1) in the presence of phosphatidylcholine which was investigated in an *in vitro* assay where digestion-protected LTP increased the release of histamine from basophils (41).

The knowledge about the oleic acid effect regarding increase of allergenicity can be used (A) in the conception of a safer immunotherapy by reduction of side effects; (B) the increase in IgE-binding has the potential of improving the sensitivity of component-resolved diagnostic tests (40).

### 2S Albumins and 7S/11S Globulins

Ara h 1, a storage protein (7 S globulin) of peanut and a major allergen as well as Sin a 2, an 11 S globulin in mustard (*Sinapis alba*) belong to the cupin superfamily. Both food allergen sources induce severe reactions. Sin a 2 has already achieved the status of a marker allergen for the severity of the reaction (42) and is responsible for cross-reactivity between mustard, peanut and tree nuts. In 2016, Angelina et al. could demonstrate one way of interaction between the globulins and lipids and the respective effects (43). In general, allergens are processed by dendritic cells (DCs) *via* endolysosomal compartments, enabling their presentation on MHC II-peptide complexes to T cells. Angelina et al. showed that mustard lipids and phosphatidylglycerol vesicles associate with Sin a 2 and diminished its uptake by DCs (43). In the same study they showed that the presence of mustard lipids together with Sin a 2 shifts the cytokine profile of DCs in a more Th2-favored direction and enhances IL-β release compared to Sin a 2 stimulation only. Further, the authors showed that in THP-1 cells mustard lipids and phosphatidylglycerol vesicles, but not peanut lipids inhibited NF-kB/AP-1 activation induced by a TLR2 ligand. Altogether, this supports the potency of these molecules to contribute to the allergic sensitization process. Further, in the same study, there is evidence that lipids also influence the direct allergen-specific activation of effector cells. The presence of mustard lipids and phosphatidylglycerol vesicles protected Sin a 2 from gastric digestion and preserved it's IgE-binding property. Phosphatidylglycerol vesicles also protected Ara h 1 from gastric digestion to a considerable extent, and 40% of the IgE-binding capacity was retained. However, Ara h 1 was not protected from intestinal digestion, but the fragment still had IgE-binding capacity for some time.

Ber e 1, a seeds storage 2S albumin in Brazil nut (*Bertholletia excelsa*), has a compact alpha-helical, disulphide-bridged rigid structure. It is hypothesized that there is a lipid-binding hydrophobic cavity, and that the allergenicity of Ber e 1 in mice depends on the presence of lipids from the Brazil nut matrix (44) as this combination stimulated murine and human CD1d-restricted iNKT cells that produced IL-4 but not IFN-γ.

The results described here make it plausible that clinically relevant single allergens pass the gastrointestinal tract in a structural condition that is still immune competent (43).

Although, Ara h 2 is a 2S albumin from peanut and shows some homology to the LTPs (both belong to the prolamin superfamily), the domain responsible for lipid-binding is not present in the Ara h 2 molecule (45). However, Ara h 2 functions as a trypsin inhibitor and is able to prevent the degradation of further accompanying allergens such as Ara h 1 (46). In addition, roasting of peanut increases the IgE-binding potential of Ara h 2 by up to 90-fold (47). This issue is thought to be attributed to the Maillard reaction which occurs during heat treatment und may lead to the creation of “neo-epitopes” on allergens (48, 49). Here, the presence of lipids, especially triacylglycerols with unsaturated fatty acids, might unintentionally lead to the creation of Maillard reaction products (MRP) as peroxidation of fatty acids generates α-dicarbonyls, highly reactive MRP precursors (50). The phenomenon of an enhanced IgE-binding after roasting is not restricted to Ara h 2 but has also been observed for other peanut allergens such as Ara h 1, Ara h 8, Ara h 12, and Ara h 13 (defensins) and Ara h 10, Ara h 11, Ara h 14, and Ara h 15 (oleosins) (12, 13, 29, 51).

### Oleosins

Oleosins are unique lipophilic allergens (20) that can be found in oil-rich seeds and plant pollen but also in mosses, ferns and algae (52, 53). They are an integral part of the phospholipid layer of oil bodies, the lipid reservoir of plants (53). Oleosins have been overlooked in their capacity of being allergens for many years due to their poor extractability and the many methodological problems that had to be faced regarding their isolation and preparation. Although some peanut allergens are known to be associated with lipids they are—in contrast to oleosins—soluble in aqueous solutions. The main characteristic of oleosins is their relatively large conserved hydrophobic domain (~7 kDa) which is anchored in a lipid storage organelle termed oil body, lipid body or oleosome. The amphipathic N-terminal and C-terminal domains of the oleosins which flank the hydrophobic domain are most often smaller and reside on the surface of the oil body to prevent their coalescence by steric hindrance and electrostatic repulsion [for a schematic model see (20)]. Based on their structural features and their tight association with lipids, oleosins are missing in aqueous-based diagnostic extracts and are yet not available as single allergens for routine diagnostic allergy tests. However, their potential to identify patients suffering from severe allergic reactions has been demonstrated in sesame allergy (2), hazelnut allergy (3), and peanut allergy (13). So far, only Schwager and co-authors managed to use oleosins purified from roasted and raw peanuts for further immunological investigations such as the basophil activation test. However, up to now it is not clear whether a potential aggregation of oleosins in an aqueous environment enhances their allergenicity through the exposure of multiple IgE-binding epitopes located in the amphipathic domains that might crosslink the Fcε receptor on basophils more efficiently.

The past years of research have raised the question whether the sensitization mechanism used by oleosins is different from that of other lipid-associated allergens. This question cannot be fully answered by the literature at present but several studies indicate that a lipid carrier-mediated mechanism is involved in the transfer across epithelial barriers (see Figure 1E) (54). Experimental data in mice suggest a more rapid uptake of substances entrapped in artificial oil bodies *via* gut and skin (55, 56). These observations might be important in many ways. First of all, oil bodies have been reported to be associated with proteins/allergens other than oleosins, and thus might act as a transfer vehicle that facilitates the contact of extrinsic allergens with immune cells (57, 58). Secondly, it has been shown that oleosins can be present even in refined oils which are typically used as ingredients in ointments and skin care products of patients with atopic eczema (59, 60). This raises the question whether the treatment of eczema patients with the respective products may put these patients at risk of being sensitized.

Palladino et al. recently published the potential “adjuvanticity” of peanut lipids with skin keratinocytes as effector cells (61). They investigated human primary keratinocytes, exposing them to all major peanut lipid classes together with or in the absence of peanut storage proteins and major allergens, Ara h 1 and Ara h 2. The peanut lipids were obtained from roasted peanuts. The group demonstrated a direct effect of peanut lipids on human keratinocytes triggering an inflammatory mediator production, because keratinocytes were able to recognize peanut lipids as exogenous stimulus. Since the pro-inflammatory mediators were elsewhere described as inducers of a barrier disruption which allows allergen penetration and subsequent allergic inflammation, the authors hypothesize a potential role of peanut lipids as adjuvant for peanut allergens (61).

## Mammalian Allergens

### Lipocalins

Members of this protein family transport small hydrophobic molecules like some lipids, steroid hormones, retinoids, pheromones, fragrances, and bilins. Lipocalins are molecules of a molecular weight between 16 and 22 kDa with different primary but similar tertiary structures. They have a binding pocket for extrinsic molecules, however, their precise role as allergens in the allergenic pathomechanism is still obscure. They are ubiquitous and can be found in arthropods, plants, and bacteria as well as mammals (62). Mammalian lipocalin allergens are carried by dander, saliva and urine. Allergens of this group consist of almost all significant inhalant mammalian allergens (mammalian dander allergens) and are distantly related to cytoplasmic fatty acid binding proteins. Allergenic representatives are *Equus caballus* (Equ c) 1 (horse), *Canis familiaris* (Can f) 1, Can f 2, Can f 4, and Can f 6 (dog), *Felis domesticus* (Fel d) 4 and Fel d 7 (cat), and *Bos domesticus* (Bos d) 2 (cattle). The dog lipocalin Can f 6 revealed considerable cross-reactivity between dog, horse, and cat. Further lipocalins have been identified in guinea pig, hamster, mouse, rat, and rabbit (63).

As such, lipocalins are not highly allergenic which may be due to the fact that most of their representatives show an amino acid identity of between 40 and 60% with endogenous human proteins which inhibits a proper immune recognition. The resemblance to the “immunological self” is thought of as one of the main reasons of low grade allergenicity (64) as they are not optimally recognized by human T cells which may favor the raise of Th2 responses.

Dog lipocalins bind LPS, thereby enhancing LPS/TLR4- signaling in for example primary macrophage-like cells (65).

β-lactoglobulin from milk, Bos d 5, is a major cows‘ milk allergen and also belongs to the lipocalin superfamily. It is known to carry hydrophobic molecules (62). Bos d 5 inserts into lipid bilayers and its interaction with phosphatidylcholine protects it from digestion in an *in vitro* gastroduodenal setting [reviewed by (22)].

## Arthropod Allergens

### Mite Protein Groups 2, 5, and 7

The HDM allergens Der p/f 2, Der p/f 5, Der p/f 7, and Der p/f 21 have lipophilic properties and are able to stimulate the immune system *via* mimicry of receptor ligands as shown in mouse models (15, 32, 66–68). Group 2 mite allergens are able to bind LPS because of their structural similarity to MD-2 (31). MD-2 is the component of the TLR4 complex that is responsible for the LPS-binding (69). Der p 2 drives TLR4 signaling followed by Th2 inflammation of the airways in wild-type mice but not TLR4-deficient mice. In this, Der p 2 acts as an autoadjuvans in the sensitization process (67). (For additional details see section heading **Lipids (and Allergens) and TLR-interaction**).

Der p 5 dimer and Der p 7 structures have hydrophobic cavities that may bind apolar ligands (15, 32). Therefore, it seems possible or even likely that this lipid-cargo-situation is synergistic between these allergens from one source in the stimulation of TLR2 (70). Der p 7 was described to bind polymyxin B, which is a bacterial lipopeptide that can bind and neutralize LPS. The crystal structure of Der p 7 reveals homology to the human lipid-binding protein family including LBP and bactericidal permeability-increasing protein (BPI) (32). This is interesting in so far as house dust mites are transporters of bacteria. However, lipid-binding studies failed to show interaction of Der p 7 to LPS or to distearoyl phosphatidyl choline (DSPC). A natural ligand for Der p 7 has not been identified yet (32).

### Cytoplasmic Fatty Acid Binding Proteins (FABPs)

#### Mite Protein Group 13

The FABP family consists of small proteins that are usually not secreted but remain in the cell cytoplasm. They are involved in the binding and transport of fatty acids in their inner cavity in vertebrates and invertebrates (71). Phylogenetic analyses revealed a potential emergence of ancestral FABP genes from lipocalins (72, 73). FABPs have been identified as allergens in diverse mites (group 13 allergens). Der p/f 13 has sequence similarities with FABPs and may reveal innate immune signaling properties *via* interaction with mite or microbial lipids (74). In their study, Satitsuksanoa et al. showed that Der p 13 is able to transport certain lipids (fatty acids) and that the proteins‘ binding to hydrophobic ligands is selective (70).

Nonetheless, their IgE-binding frequency is considered to be very low (70). This might be explained by the fact that FABPs reside inside the mite bodies and are not secreted as feces which can be more easily inhaled (75).

Nevertheless, it was shown that Der p 13 induced airway epithelial cell activation through TLR2-MyD88-NF-kappaB and MAPK-dependent mechanisms (see Figure 1D), however, the structural integrity of Der p 13 was not required suggesting an effect of the protein' s lipid cargo (70). Up to now, only the tertiary structure of Der f 13—and not of Der p 13—was solved using nuclear magnetic resonance (see Figure 1A).

### Apolipophorin

#### Mite Protein Group 14

Lipophorins are lipoproteins found in the hemolymph of most insects. Depending on the insect species they contain several apoproteins (76). Lipophorin is considered to be part of lipid bodies and transport particles of the hemolymph. Apolipophorins are poorly soluble in aqueous extracts.

Der p 14 is an LTP that bears an apolipophorin-like sequence in its N-terminal domain which shows certain similarity to the human apolipoprotein B100 and insect apolipophorins. Similar to other proteins of the apolipoprotein family, the biological function of Der p 14 seems to be the transport of lipids as it was mainly found in lipid bodies and lipid transport particles of the haemolymph in house dust mites (77). In solution, members of the group 14 HDM allergens have been found to be degraded into smaller fragments by proteolytic enzymes derived from the mite itself (e.g., group 1 allergens). However, the resulting peptides seem to be more potent in IgE-binding compared to the intact allergen and are able to trigger immune cells of allergic patients to release IL-4 and IL-13 (77, 78).

## Glycolipids: A Spectrum of Function Between Antigen and Adjuvants

Although lipids are not—like proteins—the primary target of the players of adaptive immune reactions, there is some evidence that they play a specific role in the pathomechanism of allergy by interacting with innate lymphocytes. Here we focus on the example of lipids bearing the sugar component, so called glycolipids being recognized by invariant Natural Killer T cells (iNKT).

Furthermore, since to date, there are no described glycolipids neither from peanut nor HDM, we discuss the role of iNKT cells in food allergy, based on the few available examples.

In food allergy, the immune response is evidently biased toward a phenotype dominated by Th2 cytokines. Why some food proteins induce this type of response is still a matter of debate. However, the induction of an allergic phenotype requires a primary source of IL-4. Together with basophils, iNKT cells have been identified to be the cells providing the IL-4 that determines Th2 cell differentiation and IgE production (79) and were implicated in the induction on several allergic diseases including asthma (80, 81).

In contrast to conventional protein-reactive T cells, iNKT cells recognize glycolipids as antigens presented on the MHC class-I-like molecules of the CD1 family. Asperamide B, a glycosphingolipid isolated from the ubiquitous fungus *Aspergillus fumigatus*, is the only example of a fully chemically-defined glycolipid able to induce directly allergic inflammation (independently from allergen), that resulted in airway hyperreactivity (82).

This fundamental difference and the fact that allergen sources in general contain glycolipids that can activate iNKT cells has some implications. For example, in the case of food allergy, a food-related glycolipid inducing IL-4 production by iNKT cells can act as an adjuvant able to favor the Th2 cell differentiation. In addition, while protein food allergen exposure may activate allergen-specific T cells or mast cells, the responses to exogenous (and perhaps food-related) glycolipids by iNKT cells allows the induction of allergic inflammation independently of Th2-type allergen sensitization. Moreover, the simultaneous activation of Th2 cells and iNKT cells may result in the amplification of the allergic reaction.

In a mouse model of nut allergy, the lipid-binding major allergen Ber e 1 induced specific IgE- and IgG1-antibodies only when applied together with neutral or common phospholipids derived from Brazil nuts. Antibody production was lower in NKT-deficient mice, suggesting the involvement of iNKT cells to be essential for the adjuvant activity of nut lipids (44). A recent study addressing the sensitization to one of the more frequent plant food allergens in southern Europe, Pru p 3, suggested that a lipid-ligand isolated from peach peel could act as an adjuvant for Pru p 3 *in vivo*, since the co-administration of allergen and lipid-ligand induced higher levels of IgE than allergen alone, and this effect seemed to be mediated by CD1d. In addition, the lipid-ligand was recognized by an iNKT cell line (83). Milk allergy also has a link to iNKT cell activation. Children with milk allergy had a reduced number of iNKT cells in peripheral blood in comparison to children suffering from food allergy (but not milk allergy) and healthy children. Interestingly, the remaining iNKT cells had a strong bias toward IL-4 and IL-13 production in contrast to non-allergic individuals. Further on, the glycolipid sphingomyelin present in cows' milk was identified as the responsible agent for the expansion and the IL-4 production by iNKT cells in children with milk allergy (84). A later study by the same laboratory showed that children who received oral immunotherapy (OIT) were shown to experience a reconstitution of the iNKT numbers and their switch from a Th2 toward a Th1 phenotype (85). Using a murine model, Rajavelu et al. reported that iNKT cells are required for the development of eosinophilic esophagitis driven by allergy to peanut or corn, as iNKT-deficient mice were protected. And either intravenously or intranasal administration of a potent iNKT cell agonist alone, was able to induce experimental eosinophilic esophagitis (86).

The few available studies strongly suggest that iNKT cells and glycolipids present in allergen sources play a prominent role in the pathogenesis of food allergy. This is an attractive target for food allergy therapies since interrupting the activation or effector function of iNKT cells may be highly effective in the treatment of multiple forms of food allergy, especially those where the conventional treatments are insufficient.

As stated above, glycolipids that are presented on CD1 molecules and recognized by the iNKT cell TCR have a function as antigens, but there are other lipid classes that can also activate iNKT cells while functioning as adjuvants upon stimulation of Toll-like receptors (TLRs) on antigen-presenting cells, such as DCs. Lipid-recognition through this pathway modifies the production of cytokines and provokes changes in the expression pattern and dynamics of CD1 molecules, hence influencing the presentation of glycolipids as antigens. In nature, lipids normally form mixed aggregates (micelles) when they come in contact with aqueous biological solutions, which is also the base for the formation of membranes and lipid-protein complexes. Thus, as is the case for the most sources of allergens, complex biological particles may contain glycolipid antigens for iNKT cells, physically associated with protein-allergens as “cargo” (27) and in addition to other TLR lipid-agonists (i.e., lipopeptides, lipopolysaccharide) (87). The encounter by DCs of such “lipid-allergen package” at mucosal surfaces, may deliver signals that differ in time and significance for allergic responses. For example, the immediate and purely innate signal mediated by the recognition of TLR lipid-agonists would provoke the expression of co-stimulatory molecules and the release of IL-12/IL-18, while also causing the upregulation of CD1 molecules for glycolipid presentation (88, 89). In turn, activation of iNKT cells by glycolipid antigens and/or IL-12/IL-18 would amplify the cytokine response necessary for the polarization of CD4^+^-T cells (90, 91) and the further activation of the DCs (92), providing in this way a bridge between the innate and adaptive immune response. Finally, priming *via* TLRs and cell-to-cell interaction with iNKT cells results in the maturation of DCs with an increased capacity for protein-allergen presentation on MHC class-II to conventional CD4^+^ -T cells for the purely adaptive response part of the allergic reaction. A more detailed insight of the molecular pathways involved in the lipid-TLR-iNKT axis could provide the base for additional lipid antigen-specific T cell modulation in allergic responses.

## Lipids (and Allergens) and TLR-interaction

The TLR2/TLR6 and TLR2/TLR1 heterodimers as well as TLR4 are the only known TLRs that recognize bacteria-derived lipids. These ligands are lipoproteins (93, 94) and lipopolysaccharides (95), respectively, and are naturally occurring contaminations of allergen sources like house dust mite and food. The receptors are associated with allergy and asthma, but data regarding their exact role are conflicting. There are several reviews summarizing association studies that link single nucleotide polymorphisms in TLR4 or TLR2 with a reduced risk for asthma and other allergic disorders, while other studies were not able to prove such a correlation (96–98). Further, *in vivo* mouse models showed that TLR2 and TLR4 ligands can lead to either protection or worsening of allergic symptoms, which is in strict dependency of the ligand concentration and time of administration (96, 99–101). Thus, the induction of NF-κB activation and the release of proinflammatory cytokines before sensitization may lead to an induction of regulatory responses and a shift from Th2 to Th1 responses, whereas the same cytokines in an already sensitized and allergic host can lead to an increase of the allergic symptoms.

Lipid recognition through both, TLR2 heterodimers and TLR4, differs concerning the mechanism of ligand recognition and the induced signaling cascades. In order to initiate signaling, TLR2 (93, 94, 102) needs to heterodimerize with either TLR1 or TLR6 (103, 104). The determination of which heterodimer is engaged is driven by the lipid part of the lipoproteins: in general, diacylated lipoproteins signal through TLR2/TLR6 (105) and triacylated lipoproteins signal through TLR2/TLR1 (106). Despite the differential requirements of TLR1 or TLR6 and the subsequent formation of distinct TLR2 heterodimers, the induced signaling of different lipopeptides is rather similar and dependent on MyD88 (107).

In lipopolysaccharides, the lipid part (i.e., lipid A) of the molecule is the component that drives the innate immune reaction (108). A prototypical lipid A molecule (e.g., from *Escherichia coli*) contains six fatty acids, two of which are secondary fatty acids on the first hexosamine. The binding of lipid A to MD-2 leads to a conformational change in MD-2 and the subsequent binding to TLR4 (109, 110). Interestingly, the number of acyl chains also causes TLR4-dependent species-specific differences in the immune responses toward lipid A: the 4-fold acylated precursor of lipid A is completely inactive and can be used as an antagonist in human cells, but is comparably active in mouse cells (111). The TLR4-mediated signaling is the most complex of all TLRs since both central adaptor molecules, MyD88 as well as TRIF, are involved (112–114).

Apart from the signaling pathway induced by the lipid ligands itself, some allergens can modulate TLR responses by their lipid-binding character. Herre et al. for example showed that Fel d 1 (an uteroglobin) and Can f 6 (a lipocalin) enhance ligand-induced TLR2 and TLR4 signaling, although in contrast to Der p 2, it is not by mimicking the biological function of MD-2. The authors hypothesize that both allergens can directly bind lipid ligands of TLRs and enable a more efficient transfer to cell surfaces (65). Further, when respiratory epithelial cells were stimulated with Der p 13, presumably embedding fatty acids, the production of IL-8 and GM-CSF was triggered in an TLR2-, MyD88-, MAPK-, and NF-κB- dependent signaling pathway (70).

## LTP in Lipid-Mediated Inflammation

There are not only plant-derived LTP that were shown to have considerable relevance for allergic inflammation. Several members of human LTP have a peculiar function in the immune recognition of lipids and can either enhance or reduce the immune response in the context of TLR activation. In particular the human LPS-binding protein (LBP) has been assigned a role in the development of allergic asthma in this context (115–117). LBP was found to be expressed not only in the liver, but also in the lung and the intestine (118–120), supporting organ specific activity of LBP in regulating immune responses. Although not established so far, interaction of human LTPs with hydrophobic allergens or with lipids brought as cargo of lipid-binding allergens has to be considered and is likely to shape inflammation and with this also the allergic immune response in these organs. Insights into the function of lipid-binding allergens and allergen-lipid transfer proteins in this context are rather limited so far, but are worth addressing for a better understanding of allergic inflammation. Mechanistic studies might be inspired by the knowledge of human LTPs, comprising besides LPS-binding protein (LBP): the phospholipid transfer protein (PLTP), cholesteryl ester transfer protein (CETP), bactericidal permeability-increasing protein (BPI), and the palate, lung, and nasal epithelium clone proteins (PLUNC, also called BPI-fold containing proteins, BPIF) (121, 122). Whereas, PLTP and CETP are involved in the management of endogenous lipids, the major function of BPI is in the antimicrobial defense of pathogens by binding of bacterial lipopolysaccharide (LPS) and direct antimicrobial activity (123), whereas the acute-phase protein LBP confers LPS-transport to cellular LPS receptors TLR2 and TLR4 and thereby sensitizes the immune response to minute amounts of LPS (124). All members of this family have a common hydrophobic cavity and are combined by structural homology into the tubular lipid-binding (TULIP) domain superfamily, also including the house dust mite allergens Der p 7 and Der f 7 (125, 126).

For the human CETP a tunnel mechanism was determined shuffling molecules of cholesteryl ester through a continuous tunnel formed by two interconnected hydrophobic pockets (127). LBP binds the microbial lipid LPS, and di- and triacylated lipopeptides conferring a transport to cell surface cluster of differentiation CD14 and subsequent activation of the cellular receptors TLR4/MD-2 or TLR2 in cooperation with TLR1 or TLR6 (128–130). Studies in LBP-deficient mice strongly support a role of LBP in the pulmonary immune response to infections (131, 132). The crystal structures of BPI (133) and LBP (134) revealed bound phospholipids in hydrophobic pockets distinct from the LPS binding sites of both proteins. For LBP, functional interaction with phospholipids has been shown in the context of phospholipid transport (135, 136). Moreover, phospholipid binding is of particular interest for the regulation of LBP immune functions. Inhibitory activity on LPS-mediated cell activation is reported for anionic phospholipids (137), surfactant lipids (138–140) and oxidized 1-palmitoyl-2-arachidonoyl-phosphatidylcholine (oxPAPC) (141, 142). The strong capacity of anionic lipids to reduce LBP-induced inflammation could be relevant in the case of plant allergens carrying phosphatidylglycerol lipids that are common in plant membranes (143). Recently, we demonstrated that LBP also interacts with the phospholipid membrane of immune cells. LBP is abundant on the surface of blood derived monocytes of healthy donors, and cell-associated LBP was shown to mediate a transport of LPS to intracellular compartments. Co-localization and functional studies support an involvement of LBP-mediated transport of LPS to intracellular caspases and triggering of inflammasome activation (144). These data demonstrate that phospholipid binding can interfere with the LPS-transport function of LBP, inhibit activation of TLR4 or feed LPS to intracellular receptors.

PLUNC (palate, lung and nasal epithelial clones) proteins are expressed in all epithelial surfaces of the nasopharynx, the upper airways, and the lungs (122). Besides indication for a surfactant function (145), a role of PLUNC proteins in nasal and pulmonary immune control is supported by a number of publications showing involvement in chronic rhinosinusitis (146), in the defense against *Klebsiella pneumonia* and *Pseudomonas aeruginosa* (147, 148). Expression of PLUNC proteins is upregulated by viral and bacterial infection. However, the mode of action of PLUNC proteins is not yet well-understood. The identification of lipid ligands of PLUNC proteins is ongoing research. *In vitro* binding assays showed LPS binding for some PLUNC isoforms isolated from nasal fluids (149). High affinity binding of PLUNC 1 to DPPC (dipalmitoylphosphatidylcholine) has been reported, suggesting that the major phospholipid component of pulmonary surfactant might be an endogenous ligand (150). Latherin, an allergen from horse sweat and saliva is a member of the PLUNC protein family and an inducer of IgE antibodies in horse-allergic subjects (151). The small soluble protein has an unusual surfactant activity reducing the surface tension of water that depends on the unfolding of the globular structure resulting in exposure of hydrophobic protein residues at the air-water interface (152).

## Surfactant Interaction of (Aero)Allergens

Inhaled allergens and allergen-associated lipids will eventually come in contact with the air-liquid interface of the lungs (153). The interface is formed by pulmonary surfactant which is produced and secreted by type-II bronchial epithelial cells and covers the epithelium of the airway mucosa as a lipid monolayer. Surfactant consists of a complex mixture of ~90% lipids and ~10% proteins. Phospholipids make about 80–85% of the surfactant lipid phase with the major phospholipid component dipalmitoylphosphatidylcholine (DPPC) that accounts for about 50% of the lipid mass in surfactant. Minor surfactant components are phosphatidylglycerol (PG) with 7–15% of the lipid mass and small quantities of phosphatidylinositol (PI), phosphatidylserine (PS) and phosphatidylethanolamine (PE). The hydrophobic surfactant proteins (SP), SP-B and SP-C, are closely associated with the phospholipids in the surfactant film, SP-B being essentially involved in stabilizing the surfactant structure and function by creating phospholipid lamellar bodies associated with the monolayer (154). SP-B deficiency, therefore, is lethal (155). SP-C also has a stabilizing function for the surfactant film. In *Aspergillus fumigatus*-induced allergic airway inflammation the amounts of SP-B and SP-C were both found to be strongly reduced by 50% in brochoalveolar lavage fluid (156).

The soluble surfactant proteins SP-A and SP-D are both centrally involved in the pulmonary immune response, defense to pathogens and the regulation of cellular immune functions of the alveolar macrophage (157–159). SP-A and SP-D belong to the family of collectins. The typical carbohydrate-recognition domain of these lectins preferentially recognizes carbohydrates exposed on the surface of pathogens. Recently, a role for SP-A as a local amplifier of IL-4 mediated type-2 macrophage activation in the pulmonary immune response has been discovered in the context of a helminth infection (160). This finding could have important implications for the regulation of allergic responses.

SP-A and SP-D interact with a number of allergens and interfere with the binding of IgE antibodies thus diminishing mast cell degranulation. Interaction of inhaled allergens from the house dust mite, namely Der p 1 and Der f 1, with SP-A and SP-D have been demonstrated *in vitro* and *in vivo*. Therefore, both surfactant proteins were found to bind to dust mite extracts and purified allergens (161). Interaction of Der p 1 and Der f 1 has been shown to degrade and inactivate SP-A and SP-D, thereby interfering with the natural function of the collectins in immune defense. Proteolytic fragments of SP-A and SP-D resulting from the proteinase activity of Der p 1 were less effective in binding to Der p 1, an effect that could account for the allergenicity of these proteinase mite allergens (162).

Addition of exogenous SP-A and SP-D has been demonstrated to reduce allergic hypersensitivity. A recombinant fragment of SP-D (rfhSP-D) was recently found to be effective in supressing basophil activation and histamine release to grass pollen *Phleum pratense* Phl p *in vitro* (163). Activity of SP-A and SP-D is likewise reported to reduce allergic responses to the fungal pathogen *Aspergillus fumigatus* (Asp f). Application of exogenous SP-A, SP-D, and a recombinant rfhSP-D fragment were found to reduce Asp f-induced allergic responses *in vivo* in mice (164). The structure and composition of pulmonary surfactant is strongly related to its biophysical function in the lung. Surfactant lipid composition is strictly regulated immediately after birth to enable the process of breathing (165). The physical organization of the monolayer and interconnected bilayer phases is optimized to allow surface expansion, compression, and reducing the surface tension. Phase separation of surfactant is observed in natural lung surfactant induced by the segregation of disordered phases and ordered phases such as cholesterol-containing domains (166–168). The phase behavior is important for protein surfactant interaction, as shown for the hydrophobic SP-B (169). SP-B and SP-C are both predominantly inserted in fluid disordered-like domains of surfactant membranes (170).

Changes in lung surfactant composition can have a great impact on the biophysical surfactant function. Low amounts of bacterial LPS have been shown to disturb monolayers made from DPPC or a surfactant-like lipid mixture made from DPPC, 16:0/18:1 phosphatidylglycerol (POPG), and palmitic acid. Intercalation of LPS into the monolayer resulted in fluidization of the lipid phase, promoting early collapse and preventing the attainment of high surface pressure (171). The input of LPS carried on many allergens should therefore also be considered with respect to its effects on the biophysical surfactant function. Similar effects might be expected upon inhalation of hydrophobic allergens or lipid-binding allergens, which will naturally interact with the surfactant lipid phase due to hydrophobic interaction. An example for direct allergen surfactant interaction is reported for the aeroallergen Ole e 1 from olive tree (172). The hydrophobic N-glycosylated protein Ole e 1 showed interfacial absorption at an air-water interface, leading to an increase in surface pressure. Application of Ole e 1 to the aqueous phase under DPPC monolayer membranes led to membrane interaction of the protein resulting in an increase in surface pressure. These experiments also indicated a change in the protein orientation or conformation of the allergen upon membrane insertion, which might induce protein oligomerization or aggregation. Such structural changes are likely to affect the allergenic properties. Thus, an increase in IgE-binding and allergenicity was demonstrated for dimerized birch pollen allergen Bet v 1 (173). Another interesting aspect of the study was the observation, that Ole e 1 also interacted with 18:1(Δ9-cis) phosphatidylcholine (DOPC): sphingomyelin (SM): cholesterol model membranes, mimicking the plasma membrane of eukaryotic cells. This interaction was preferentially observed in liquid-ordered (lo) cholesterol-rich domains, indicating that membrane interaction should also be considered as a mechanism to bring hydrophobic allergens in direct contact with epithelial cells of the alveolar lining *via* interaction with lipid raft domains.

On the basis of these data, interaction of larger hydrophobic allergen/lipid aggregates such as oleosins appears to be likely considering the flexibility and connectivity of the surfactant phase and should certainly be addressed in future studies. Besides the impact on the allergic response and inflammatory regulation, surfactant interaction of lipophilic (aero)allergens leading to changes of the surfactant phase and organization is likely to have biophysical consequences that could affect lung function in general.

## Potential Effects of Allergen-Lipid-Binding on the Development of Asthma and Allergy

Up to now, the evidence for an association between single allergenic molecules and disease development and phenotype is epidemiological and based on the still incomplete component-resolved diagnostic tests.

For years, food storage proteins and their structural characteristics were in part responsible for the more severe class I food allergy when compared with the pollen-associated class II allergy, arguing for the fact that storage proteins are heat and digestion resistant and therefore are stable enough to induce severe symptoms.

The more we learn, however, on single allergenic molecules, the more complex the picture gets, and there is still a growing body of evidence that indeed single allergenic molecules play an important role not only in disease development but also in the shaping of phenotypes.

*Via* component-allergy diagnostic tests with an increasing number of single allergens from different food and inhalant allergen sources investigations on the association between clinical phenotype and sensitization profile are performed and have in some cases already resulted in the identification of potential marker allergens. Some success in this regard has been achieved for allergens from the protein families described in this manuscript. Presently, the lipocalins, Can f 1, Can f 2, and Can f 5 are available for component-resolved diagnostics as species-specific marker allergens (62). There is an association of lipocalin sensitization with asthma in children (174–176).

Whereas plant storage proteins can be used as indicators for severe food allergy, those allergens that cross-react with tree pollen in general represent sensitization that is only associated with mild to moderate symptoms. Some examples are already mentioned here in the context of nut allergy. For peanut allergy, the storage protein Ara h 2 already has been appointed as marker allergen for severe reactions. If patients have an anti-Ara h 2-IgE concentration of >42.2 kU/L, they will develop severe symptoms in 95% of cases (177). Interestingly, sensitization to peanut storage proteins only seems to occur if peanut allergy started in childhood which makes these allergens potential markers for early onset peanut allergy.

Peanut oleosins were recently shown to be marker allergens for the severity of the reaction to peanuts as well (13). Different subgroups of peanut-allergic patients were investigated: 53/74 experienced severe reactions, *n* = 52 had a genuine peanut allergy and had IgE to oleosins. Only one individual with severe reactions was a patient with a pollen-associated peanut allergy. This patient was anti-oleosin-IgE negative. All those with mild symptoms were patients with a pollen-associated peanut allergy (20). The fact that oleosins come along surrounded by phospholipids directed our research to allergen-lipid-interactions and its clinical impact.

The only oleosin-IgE-negative patient with shock to peanuts in our study was Ara h 8-IgE-positive, meaning he had a pollen-associated peanut allergy, and IgE to all other severity marker allergens were negative. This observation is supported by Glaumann et al. who described a shock in a patient who had consumed 300 g of roasted peanuts instead of a proper meal and only had Ara h 8-specific IgE antibodies (178). It is known from further examples of Bet v 1-homologs that these labile allergens, which are easily destroyed by digestion and thermal processing, may induce severe reactions when ingested in high concentrations. It is probable that in the case of Ara h 8 its association with lipids protects the allergen from immediate destruction after peanut consumption. We could show some years ago in a time course experiment, that Ara h 8 is associated with lipids which protected it for a certain period of time from enzymatic digestion (12) (see Figure 1B).

Mite allergy is a well-established risk factor for the development of asthma during childhood, and single mite allergens have been investigated in detail with regard to their differential influence in pathogenesis. Coming from the clinical observation, well-characterized subgroups of patients were tested for their individual sensitization profile. Investigations on the sensitization pattern to single HDM allergens, which are basically epidemiological studies, had revealed that among all those already purified and structurally characterized mite molecules, lipophilic allergens show a stronger association with asthma (6, 7). Recombinant Der p 2, Der p 5, and Der p 7 are significantly more often recognized by asthmatic patients. rDer p 5 was 3-times more often recognized by asthmatic children than by non-asthmatic children, and patients with IgE-reactivity to rDer p 5 had a probability of 85% of having HDM-asthma (7). Der p 1 or Der p 23 can predict asthma at school age (179). Cytoplasmic FABP allergens are restricted to mite bodies and are not transported *via* mite feces. Therefore, they are not important for the induction of lung/bronchial reaction due to their size but most probably responsible for HDM-induced skin reactions since they are major allergens for individuals with atopic dermatitis (180), thereby promoting organ-specificity of single allergens.

However, not only single inhalant allergens reach the broncho-alveolar cells. This seems to be the case for some food allergens as well, and not just as “pollen-associated” food allergy. Several patients with severe peanut allergy describe that they develop an allergic bronchoconstriction whenever someone in the same room opens a tin of peanuts (which was one main reason to forbid peanut snacks on airplanes where allergens were obviously distributed *via* air conditioning). Roberts et al. showed that a subgroup of food allergic children developed asthma when exposed to the aerosolized form of the food, for example while it was cooked in their presence. The authors confirmed that effect *via* bronchial provocation (181) demonstrating that foods can behave as aeroallergens. Investigations with a chewing simulator for food breakdown and flavor compound release allows deeper insight in the complex processing of food in the mouth where fluids and gas act together (182). It therefore can be hypothesized that the act of chewing peanuts drives allergens as aerosols into the bronchial system. This hypothesis is supported by the knowledge, that the delivery of the allergens takes place on the particles, containing allergens, and other chemically different substances, such as lipids. These particles become airborne and have a size small enough to reach the lungs (183). Additionally, such particles will face the milieu of the lungs, composed not only of immune cells but pulmonary surfactant as well. In this compartment, allergens/lipids come into contact with the surfactant-containing air-liquid interface that covers the epithelium of the airway mucosa underneath (153), as discussed in detail above [see section Surfactant Interaction of (Aero)allergens].

Overall, the recent evidence for the induction of pro-inflammatory responses of skin keratinocytes by peanut lipids and the facilitation of allergen penetration of the skin barrier by lipids, together with other potential lipid-associated transport mechanisms for allergens across barriers might be an explanation for a transcutaneous sensitization to peanuts (61).

Single allergens can be used as tools for mechanistic research to clarify sensitization and allergy elicitation, thereby allowing for pathomechanistic insights with the consequence of a development of new therapeutic approaches including the development of new adjuvants and treatment vectors. Allergens with lipophilic features are missing in clinically relevant peanut extracts and most probably are underrepresented in diagnostic and therapeutic solutions of house dust mites (184), since isolation and purification methods and strategies are particularly difficult (4).

As described in more detail in the allergen-section of this manuscript, the allergen-lipid-interaction of single allergens is rather different and their effect on several cells in different compartments diverse. It is plausible that dependent on the mode of interaction of the different lipophilic allergens with lipids, these lipids may participate in the pathomechanism of asthma and allergy by (a) serving as (additional) antigen, (b) adjuvant, and/or (c) precursor for a Th2-dominated milieu.

Altogether, HDM and peanut allergy appear as good models not only for studying the effects of allergen-lipid-interactions on disease development *via* the sensitization to distinct single allergens but also for the correlation between single allergens and symptom development at specific organs (organ specificity).

## Author Contributions

UJ designed and wrote the manuscript. AS, KD, KS, HH, NG, and CS wrote the manuscript. CS created Figure 1. CS and UJ created Table 1.

### Conflict of Interest Statement

The authors declare that the research was conducted in the absence of any commercial or financial relationships that could be construed as a potential conflict of interest.

## Footnotes

^1^Available online at: www.allergen.org Official Homepage of the WHO/IUIS Allergen Nomenclature.
